# Endometrial scratching in women with implantation failure after a first IVF/ICSI cycle; does it lead to a higher live birth rate? The SCRaTCH study: a randomized controlled trial (NTR 5342)

**DOI:** 10.1186/s12905-017-0378-y

**Published:** 2017-07-21

**Authors:** N. E. van Hoogenhuijze, H. L. Torrance, F. Mol, J. S. E. Laven, E. Scheenjes, M. A. F. Traas, C. Janssen, B. Cohlen, G. Teklenburg, J. P. de Bruin, R. van Oppenraaij, J. W. M. Maas, E. Moll, K. Fleischer, M. H. van Hooff, C. de Koning, A. Cantineau, C. B. Lambalk, M. Verberg, M. Nijs, A. P. Manger, M. van Rumste, L. F. van der Voet, A. Preys-Bosman, J. Visser, E. Brinkhuis, J. E. den Hartog, A. Sluijmer, F. W. Jansen, W. Hermes, M. L. Bandell, M. J. Pelinck, J. van Disseldorp, M. van Wely, J. Smeenk, Q. D. Pieterse, J.C. Boxmeer, E.R. Groenewoud, M. J. C. Eijkemans, J. C. Kasius, F. J. M. Broekmans

**Affiliations:** 1 University Medical Center Utrecht, Heidelberglaan 100, 3584 CX Utrecht, The Netherlands; 2 Academic Medical Center Amsterdam, Amsterdam, The Netherlands; 3 Erasmus Medical Center Rotterdam, Rotterdam, The Netherlands; 4 Gelderse Vallei Hospital, Ede, The Netherlands; 5 Gelre Hospital, Apeldoorn, The Netherlands; 6 Groene Hart Hospital, Gouda, The Netherlands; 7 Isala Fertilityclinic, Zwolle, The Netherlands; 8 Jeroen Bosch Hospital, Den Bosch, The Netherlands; 9 Maasstad Hospital, Rotterdam, The Netherlands; 10 Maxima Medical Center, Veldhoven, The Netherlands; 11 Onze Lieve Vrouwe Gasthuis, Amsterdam, The Netherlands; 12 Radboud University Medical Center, Nijmegen, The Netherlands; 13 St. Franciscus Gasthuis, Rotterdam, The Netherlands; 14 Ter Gooi Hospital, Hilversum, The Netherlands; 15 University Medical Center Groningen, Groningen, The Netherlands; 16 Vrije Universiteit Medical Center, Amsterdam, The Netherlands; 17 Fertility Clinic Twente, Hengelo, The Netherlands; 18 Nij Geertgen, Elsendorp, The Netherlands; 19 Diakonessenhuis, Utrecht, The Netherlands; 20 Catharina Hospital, Eindhoven, The Netherlands; 21 Deventer Hospital, Deventer, The Netherlands; 22 Alrijne Hospital, Leiderdorp, The Netherlands; 23 Amphia Hospital, Breda, The Netherlands; 24 Meander Medical Center, Amersfoort, The Netherlands; 25 Maastricht University Medical Center, Maastricht, The Netherlands; 26 Wilhelmina Hospital Assen, Assen, The Netherlands; 27 Leiden University Medical Center, Leiden, The Netherlands; 28 Medical Center Haaglanden-Bronovo-Nebo, The Hague, The Netherlands; 29 Albert Schweitzer Hospital, Sliedrecht, The Netherlands; 30 Scheper Hospital, Emmen, The Netherlands; 31 St. Antonius Hospital, Nieuwegein, The Netherlands; 32 Dutch Consortium for Healthcare Evaluation and Research in Obstetrics and Gynecology – NVOG Consortium 2.0, Dutch, The Netherlands; 33 St. Elisabeth-Twee Steden Hospital, Tilburg, The Netherlands; 34 Haga Hospital, The Hague, The Netherlands; 35 Reinier de Graaf Gasthuis, Delft, The Netherlands; 36 Noordwest Ziekenhuisgroep, Den Helder, The Netherlands

**Keywords:** Endometrial injury, Endometrial scratching, Endometrial scratch, In vitro fertilization, Intracytoplasmic sperm injection, IVF, ICSI, Implantation failure

## Abstract

**Background:**

Success rates of assisted reproductive techniques (ART) are approximately 30%, with the most important limiting factor being embryo implantation. Mechanical endometrial injury, also called ‘scratching’, has been proposed to positively affect the chance of implantation after embryo transfer, but the currently available evidence is not yet conclusive. The primary aim of this study is to determine the effect of endometrial scratching prior to a second fresh in vitro fertilization/intracytoplasmic sperm injection (IVF/ICSI) cycle on live birth rates in women with a failed first IVF/ICSI cycle.

**Method:**

Multicenter randomized controlled trial in Dutch academic and non-academic hospitals. A total of 900 women will be included of whom half will undergo an endometrial scratch in the luteal phase of the cycle prior to controlled ovarian hyperstimulation using an endometrial biopsy catheter. The primary endpoint is the live birth rate after the 2^nd^ fresh IVF/ICSI cycle. Secondary endpoints are costs, cumulative live birth rate (after the full 2^nd^ IVF/ICSI cycle and over 12 months of follow-up); clinical and ongoing pregnancy rate; multiple pregnancy rate; miscarriage rate and endometrial tissue parameters associated with implantation failure.

**Discussion:**

Multiple studies have been performed to investigate the effect of endometrial scratching on live birth rates in women undergoing IVF/ICSI cycles. Due to heterogeneity in both the method and population being scratched, it remains unclear which group of women will benefit from the procedure. The SCRaTCH trial proposed here aims to investigate the effect of endometrial scratching prior to controlled ovarian hyperstimulation in a large group of women undergoing a second IVF/ICSI cycle.

**Trial registration:**

NTR 5342, registered July 31^st^, 2015.

**Protocol version:**

Version 4.10, January 4th, 2017.

## Background

In 2005, the combined number of assisted reproductive technique (ART) cycles in the USA, Australia, New Zealand and Europe was more than 600 000, and in 2002 the estimated global number of started ART cycles was 1 million [1]. In-vitro fertilization (IVF) and intracytoplasmic sperm injection (ICSI) are the most common ART treatments. Despite advances that have been made since the introduction of these techniques, implantation of the embryo is still the most important rate-limiting step: even high quality embryos frequently fail to implant resulting in an implantation rate of approximately 25–30% per transferred embryo [2, 3]. Over the past decade, intentional injury to the endometrial lining, also called “scratching”, has been proposed as a method to improve implantation. The foundation for these findings was laid early in the 20^th^ century, when Loeb et al. showed that in guinea pigs, mechanical irritation of the endometrium at 2–9 days after ovulation led to decidualization [4]. Barash et al. [5] were the first to test the hypothesis that endometrial injury in the natural cycle prior to controlled ovarian hyperstimulation (COH) could increase the chance of pregnancy, and found a two-fold increase in live birth rate after multiple endometrial scratches compared to no scratch. Since then, multiple study groups have investigated the effect of endometrial scratching on implantation rate with differences in population, device used (a soft plastic endometrial biopsy catheter, Karman cannula or Novak curette), timing (luteal or follicular phase), and frequency [6–16]. Several hypotheses supporting the positive effect of scratching on pregnancy rates have been proposed but the exact mechanism remains unclear [17, 18]. A Cochrane review by Nastri et al. suggests that for women undergoing ART, an endometrial scratch in the month prior to COH improves the chance of achieving a clinical pregnancy and live birth in women with two or more previously failed embryo transfers, but the evidence is of moderate quality at best [17]. Another review on endometrial injury in women undergoing ART could not perform a meta-analysis due to significant clinical heterogeneity, and could therefore not draw conclusions on the effectiveness of scratching [18]. This stresses the need for large randomized controlled trials on specific subgroups of subfertile couples. The study introduced here aims to determine if endometrial scratching in the cycle prior to COH in women with one previously failed full IVF/ICSI cycle increases the chance of live birth. It also aims to evaluate if this leads to a decrease in the number of ART cycles and costs needed to achieve a live birth. Furthermore, the study contains an embedded study that aims to determine characteristics, such as RNA profiles, of the endometrial lining that are associated with implantation failure.

## Method

### Study objective & design

The aim of this study is to evaluate whether endometrial scratching in the cycle prior to a second ART cycle leads to a higher number of ongoing pregnancies with live births as compared to no scratching, in women with a failed first IVF/ICSI cycle. While other studies have chosen to study women with recurrent implantation failure (RIF), usually defined as implantation failure of three high-quality embryo transfers, this study focuses on an intervention after one failed IVF/ICSI cycle in order to augment the efficacy of ART in an early phase of treatment. Moreover, the study seeks to determine endometrial characteristics such as RNA profiles correlating with (un)successful implantation.

The SCRaTCH study is a Dutch multicenter – both academic and non-academic – randomized controlled trial (RCT) coordinated from the University Medical Center Utrecht, and will be performed within the Dutch Consortium for Healthcare Evaluation and Research in Obstetrics and Gynecology. A prospective cohort study is embedded within the RCT and will be performed in 6 dedicated centers, in which the endometrial biopsy will be banked after which characteristics associated with (un)successful implantation will be determined. The primary endpoint is live birth rate after the second fresh ART cycle. Secondary endpoints include cumulative ongoing pregnancy and live birth rate after the full second ART cycle; cumulative ongoing pregnancy and live birth rate after 12 months of follow-up; biochemical, clinical, ongoing and multiple pregnancy rate; implantation rate; miscarriage rate; time to pregnancy; costs; and endometrial tissue parameters associated with implantation failure, such as endometrial gene expression profiles.

In the RCT, a total of 900 women will be included of which approximately 200 will also be included in the embedded study. Based on previous studies, the difference in live birth rate after the second ART cycle is estimated to be at least 9% between the patients with and without endometrial scratch (39% vs. 30%). The number of patients needed to have 80% power (with two-tailed alpha of 0.05) to detect such a difference is 450 per study arm, resulting in 900 patients in total. This number takes into account an estimated dropout rate of 3% and is calculated without continuity correction in STATA.

Monitoring will be performed by the Data and Safety Monitoring Board (DSMB) of the Dutch Consortium for Healthcare Evaluation and Research in Obstetrics and Gynecology – NVOG 2.0. The DSMB has several roles, including monitoring for evidence of treatment harm, such as serious adverse events, and advising on continuation or terminating of the trial. Interim analyses have not been planned. In case of injury as a consequence of study participation, compensation is assured through a liability insurance of the coordinating center (the sponsor). The DSMB charter is shown in the appendix.

### Study population

Women planning a second full IVF/ICSI cycle with a regular indication for IVF/ICSI and a failed implantation after one full fresh ART cycle are eligible. At least one embryo (either fresh or frozen/thaw) must have been transferred during the first IVF/ICSI cycle. Failed implantation is defined as the absence of a clinical pregnancy in the full first cycle. In case of total fertilization failure after the first IVF/ICSI cycle, and at least one embryo transfer during the second IVF/ICSI cycle, patients are also eligible before the start of their third IVF/ICSI cycle. Inclusion criteria are female age between 18 and 44 years, with primary or secondary infertility, and a normal transvaginal ultrasound defined as no visible intracavitary pathology (e.g. polyps or intramural myomas with impression of the uterine cavity). Exclusion criteria are a history of lower abdominal or pelvic infection, a higher chance of intra-abdominal infection due to intestinal surgery, endometriosis grade 3 and 4, previous caesarean section with niche formation, the presence of untreated unilateral or bilateral hydrosalpinx, previous endometrial scratching, meno-metrorrhagia, and untreated endocrine abnormalities. Patients undergoing oocyte donation cycles or preimplantation genetic diagnosis (PGD) are also excluded since oocyte donation patients have a different etiology for infertility than recurrent implantation failure, and PGD patients usually are not infertile.

### Recruitment, consent and randomization

Eligible women will receive oral and written information from their gynecologist or fertility physician, and will be invited for additional counseling by the investigator or research nurse to allow the women to make an informed decision on participating in the study. After signing a written informed consent the patient is randomly allocated to either the scratch procedure before the start of the second IVF/ICSI cycle or to direct start of the second IVF/ICSI cycle. Randomization is performed by a web-based randomization program using random blocks with block size varying from two to four. Due to the nature of the intervention, patients and physicians are not blinded for this study, nor will a placebo intervention be performed. As the primary outcome is a ‘hard endpoint’ we do not expect results to be influenced by the fact that there is no allocation concealment.

### Study procedure

In the intervention group, an endometrial scratch will be performed once during the luteal phase of the cycle prior to controlled ovarian hyperstimulation. The scratch will be done 10 to 5 days prior to the expected menstruation in the overall group, and 5-8 days after a positive urine ovulation test for the subgroup participating in the embedded study. A dedicated gynecologist or fertility physician, with a maximum of 3 dedicated physicians per center, will perform the scratch. Women are instructed not to be pregnant during the procedure. After disinfection of the uterine cervix with betadine, an endometrial biopsy catheter will be introduced through the cervix up to the uterine fundus. The piston will be drawn back to the end of the biopsy cannula, after which the examiner slowly retracts the catheter while rotating over several ranges of 360° during 1–2 min. For the subgroup of whom the endometrial biopsy will be stored, the protocol differs slightly. Firstly, during the procedure a sterile gown, gloves and cap will be worn in order to prevent RNA contamination. Secondly, disinfection will not be performed with betadine but with sterile water because betadine could affect the outcomes of RNA analysis. Directly after the procedure, sterile gloves will be changed for new gloves in order to prevent RNA contamination. The endometrial tissue will be divided into three equal parts, put into Tissue Sampling Storage Tubes (3 ml, Cat. No. 68–4000-00, Fluid X) and snap frozen in liquid nitrogen. Time from taking the biopsy to snap freezing the tissue is maximally 3 min. Subsequently, tissue will be stored in freezers at -80 °C.

**Fig. 1:**
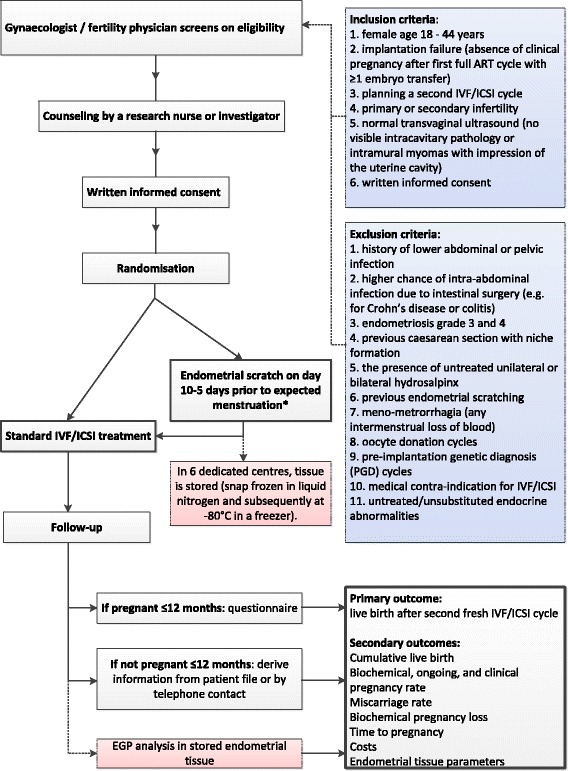
Study flowchart

Immediately after taking the biopsy, the patient will be asked to indicate the degree of discomfort on a visual analog scale. One week after the procedure the patient will be contacted to evaluate if any complications occurred. All patients, both the intervention and control group, will receive standard ART therapy consisting of a short antagonist, short agonist, or long agonist protocol. GnRH agonists can be used in the overall group, but within the embedded study subpopulation GnRH agonists are started after the scratch has been performed. An overview of the phases of the study is shown in Fig. 1.

ART, assisted reproductive techniques. IVF, in vitro fertilization. ICSI, intracytoplasmic sperm injection. EGP, endometrial genomic profile. *Timing applies to the overall study. In the embedded study the endometrial scratch is performed on day 5–8 after the LH surge measured by urine ovulation tests. The boxes in pink apply to the embedded study.

### Follow-up

Each center registers if a patient has conceived during the 12 months after randomization. When a woman has become pregnant, she will undergo ultrasounds at approximately 7 weeks and 10–12 weeks of gestation, after which she will continue prenatal care at the midwife or the gynecologist. At the due date, a questionnaire will be sent with questions about the course of the pregnancy and the delivery. If a woman has not conceived within 12 months, information on further fertility treatment will be extracted from the electronic patient file. Missing information will be completed by telephone contact with the participant, using a structured questionnaire.

### Statistical analysis

Anonymized data will be collected in a web based registration system. Database cleaning will be carried out by internal consistency checks and identification of database entries outside expected ranges. Analysis will primarily be according to intention-to-treat. If many women in the intervention group end up not having the scratch procedure, a per protocol analysis may also be performed to clarify whether the magnitude of effect may have been underestimated.

SPSS and Excel will be used to perform the statistical analysis. The outcome variables of the primary and secondary endpoints (except the costs, of which analysis is described below) will be compared between the treatment arms, and expressed as relative risk with 95% confidence interval. A probability (p) less than 0.05 will be considered to be significant.

### Cost-effectiveness analysis

The economic evaluation is designed from both a healthcare and a societal perspective. A cost-effectiveness analysis covering a period of 12 months will be performed parallel to the clinical trial. This cost-effectiveness analysis will be based on live birth rate and average costs per patient. Impact on patient’s life will be expressed in quality-adjusted life years (QALY), and will be estimated based on other studies performed within the Dutch Consortium for Healthcare Evaluation and Research in Obstetrics and Gynecology – NVOG 2.0. A decision model will be used to evaluate the optimal strategy, taking into account the time to pregnancy, direct and indirect costs on the one hand, and estimated QALY on the other hand. Moreover, the incremental cost-effectiveness ratio (ICER) and long term costs such as delivery and perinatal costs will be determined. Sensitivity analysis will define robustness of the results.

## Discussion

Intentional endometrial injury is frequently being performed in women undergoing IVF without conclusive scientific evidence on its beneficial effects. For example, across Australia, New Zealand and the UK it is advised to patients undergoing IVF by 83% of the clinicians working in a fertility clinic [19]. Multiple studies have been performed to investigate the effect of endometrial scratching in women undergoing ART cycles, but the method of scratching, the population being scratched and the study quality varies widely [17, 18]. Due to this heterogeneity in design and variability in quality, it remains unclear for whom this extra treatment could be beneficial, and for whom it is redundant [18]. This stresses the need for large randomized controlled trials in specific subpopulations and for an individual patient data analysis to reduce heterogeneity. The current study will evaluate the effect of endometrial scratching on live birth after the second fresh ART cycle in women with a failed first full IVF/ICSI cycle. Although this subgroup is rather well-specified, heterogeneity still exists: number of other fertility treatments such as intra-uterine insemination prior to IVF could differ, and women may have had a different number of frozen/thaw embryo transfers. However, from an intention-to-treat point of view, this study has selected a clear population; namely a population that has shown to have had at least one failed implantation. Although frequently the term ‘implantation failure’ is used for women with implantation failure of at least three high-quality embryo transfers [20, 21], this study focuses on maximizing the effectiveness of ART earlier during the process, keeping in mind both patient burden and healthcare costs. In addition, Koot et al. have shown that endometrial genomic profiles (EGP) show different characteristics in women with and without repeated implantation failure (RIF) [22]. The current study seeks to identify if this EGP predicts the chance of conception within 1 year of IVF treatment. Also, identifying an EGP that is related to endometrial failure could help clinicians in counselling patients and it opens possibilities for further research into endometrial receptivity. Another point of discussion is whether the use of povidone-iodine during the endometrial scratch could affect implantation. To our knowledge, no RCTs have been performed investigating the effect of cervical disinfection with povidone-iodine 3–4 weeks prior to embryo transfer on the endometrial lining or on embryo implantation. Therefore, it was decided that in this trial, the benefit of reducing the risk of infection outweighs the risk of a possible negative effect of povidone-iodine on embryo implantation. Still, for the subgroup of the embedded study povidone-iodine disinfection is not used because this could influence the outcome of RNA profile analysis. In conclusion, the SCRaTCH study is a large randomized controlled trial and aims to elucidate if an endometrial scratch in the luteal phase prior to COH improves the chance of a live birth for patients with a failed first IVF/ICSI cycle.

## Abbreviations

ART: Assisted reproductive techniques
COH: Controlled ovarian hyperstimulation
EGP: Endometrial genomic profile
ICER: International cost-effectiveness ratio
ICSI: Intracytoplasmic sperm injection
IVF: In vitro fertilization
PGD: Preimplantation genetic diagnosis
RCT: Randomised controlled trial
RIF: Recurrent implantation failure
RNA: Ribonucleic acid
